# Cilostazol as an add-on therapy for patients with Alzheimer’s disease in Taiwan: a case control study

**DOI:** 10.1186/s12883-017-0800-y

**Published:** 2017-02-23

**Authors:** Shu-Yu Tai, Chun-Hung Chen, Chen-Yu Chien, Yuan-Han Yang

**Affiliations:** 1 Department of Family Medicine, School of Medicine, College of Medicine, Kaohsiung Medical University, Kaohsiung City, Taiwan; 2 Department of Family Medicine, Kaohsiung Medical University Hospital, Kaohsiung Medical University, Kaohsiung City, Taiwan; 3 Department of Family Medicine, Kaohsiung Municipal Ta-Tung Hospital, Kaohsiung Medical University Hospital, Kaohsiung Medical University, Kaohsiung City, Taiwan; 4 Research Center for Environmental Medicine, Kaohsiung Medical University, Kaohsiung City, Taiwan; 5 Department of Neurology, Kaohsiung Municipal Hsiao-Kang Hospital, Kaohsiung Medical University, Kaohsiung City, Taiwan; 6 Department of Neurology, Kaohsiung Medical University Hospital, Kaohsiung City, Taiwan; 7 Department of Otorhinolaryngology, School of Medicine, College of Medicine, Kaohsiung Medical University, Kaohsiung City, Taiwan; 8 Department of Otorhinolaryngology, Kaohsiung Medical University Hospital, Kaohsiung Medical University, Kaohsiung City, Taiwan; 9 Department of Neurology, Kaohsiung Municipal Ta-Tung Hospital, Kaohsiung City, Taiwan; 10 Department of and Master’s Program in Neurology, Faculty of Medicine, Kaohsiung Medical University, HospitalNo.100, Tzyou 1st Road, Kaohsiung 807, Taiwan

**Keywords:** Cilostazol, Alzheimer’s disease, MMSE, CDR-SB

## Abstract

**Background:**

Combination therapy using acetylcholinesterase inhibitors (AChEIs) and cilostazol is of unknown efficacy for patients with Alzheimer’s disease (AD).

**Methods:**

We explored the therapeutic responses by using a case–control study, which was conducted in Taiwan. We enrolled 30 participants with stable AD who were receiving cilostazol (50 mg) twice per day as an add-on therapy combined with AChEIs, and 30 participants as controls who were not receiving cilostazol as an add-on therapy. The therapeutic responses were measured using neuropsychological assessments and analyzed in relation to cilostazol use, apolipoprotein E genotype, and demographic characteristics. Mini-mental state examination (MMSE) and clinical dementia rating sum of boxes (CDR-SB) were administered at the outset of the study and 12 months later. Multiple logistic regression analysis was used to estimate the association between the therapeutic response and cilostazol use.

**Results:**

For the therapeutic indicator of cognition, Cilostazol use (adjusted odds ratio (aOR) = 0.17, 95% confidence interval (CI) = 0.03–0.80), initial CDR-SB score (aOR = 2.06, 95% CI = 1.31–3.72), and initial MMSE score (aOR = 1.41, 95% CI = 1.11–1.90), but not age, sex, education, or ApoE ε4 status, were significantly associated with poor therapeutic outcomes. For the therapeutic indicator of global status, no significant association was observed between the covariates and poor therapeutic outcomes.

**Conclusions:**

Cilostazol may reduce the decline of cognitive function in stable AD patients when applied as an add-on therapy.

## Background

More than 35 million people worldwide [1] and 5.5 million people in the United States have Alzheimer’s disease (AD). AD causes deterioration of the memory and other cognitive domains, thereby increasing morbidity and mortality in the elderly population [2].

AD is the most common form of dementia, accounting for 50–56% of cases at autopsy and in clinical series. In addition, AD combined with intracerebral vascular disease accounts for another 13–17% of cases. The principal risk factor for AD is advanced age. The incidence of AD doubles every 5 years after 65 years of age, with 1275 new cases diagnosed per year per 100, 000 people aged 65 years or more [3, 4]. Moreover, AD is one of the most common causes of disability in the elderly population [2], thereby having a considerable effect on caregivers. In 2010, dementia resulted in approximately 486,000 deaths [5]. In developed countries, AD has one of the highest economic burdens of all diseases [6].

No cure currently exists for AD [7, 8]. Acetylcholinesterase inhibitors (AChEIs) are often used and may be beneficial in mild-to-moderate cases. However, their overall benefit may be minor [9]. New treatment modalities for AD such as immunotherapy, γ-secretase inhibitors, and cerebral circulation attenuation are promising [10].

Cilostazol is a selective inhibitor of cyclic nucleotide phosphodiesterase 3 (PDE3). Cilostazol possesses many pharmacological activities including anti-inflammatory, antioxidative, and antiapoptotic effects in the brain [11]. In addition, cilostazol prevented cerebral hypoperfusion-induced cognitive impairment and white matter damage in a rat model by the occlusion of the common carotid artery through bilateral ligation [12]. A more recent preliminary study involving 10 patients with moderate AD demonstrated that a combination therapy of donepezil and cilostazol significantly improved Mini-Mental State Exam (MMSE) scores and maintained the status of participants unchanged until the end of the follow-up period [13]. Cilostazol may prevent the neuronal death and cognitive impairment caused by AD. However, the sample size in the study was small (10 patients) and the follow-up period was relatively short (an average of 6–7 months). In addition, the efficacy of only the combination therapy of donepezil and cilostazol was evaluated.

In the current study, we examined the therapeutic response to cilostazol as an add-on therapy in patients with stable AD receiving AChEIs in Taiwan. We hypothesized that cilostazol, which is a phosphodiesterase 3 inhibitor, would improve cognitive decline in patients with AD.

## Methods

### Patients

To investigate the effect of cilostazol as an add-on therapy in patients with stable AD receiving AChEIs, we conducted a case–control study in the regional teaching hospital of Kaohsiung Municipal Ta-Tung Hospital in southern Taiwan.

We included 30 patients with stable AD receiving cilostazol (50 mg) twice per day as an add-on therapy with AChEIs for at least 12 months between December 2014 and December 2015 in the study group. The patients with stable AD were defined as those with satisfactory medication adherence, 30 days or fewer without AChEIs in the past year, and follow-up visit compliance of at least 80%. No adjustment of the dosage of AChEIs or use of any other add-on anti-dementia medication was allowed during the study period. We matched each study patient with one patient with stable AD receiving AChEIs for at least 12 months, applying their age (within 2 years) and educational level as a control. The patients in the control group did not receive cilostazol (50 mg) as an add-on therapy.

The AD diagnosis was based on the National Institute of Neurological and Communicative Disorders and Stroke and the Alzheimer's Disease and Related Disorders Association criteria [14], which include a series of comprehensive neuropsychological tests such as the MMSE [15], Cognitive Assessment Screening Instrument (CASI) [16], Neuropsychiatric Inventory [17], and Clinical Dementia Rating Sum of Boxes (CDR-SB) [18]. Patients with other conditions possibly contributing to their AD diagnosis were excluded (including nutritional deficiency, metabolic encephalopathy, hypothyroidism, toxin, and CNS infection or tumor).

### Evaluation

Various neuropsychological assessments including the MMSE, CASI, and CDR-SB were performed initially and every 6 months thereafter with all patients to evaluate their therapeutic response to the AChEIs. These assessments were performed by a senior neuropsychologist and an experienced physician based on information provided by a knowledgeable collateral source (usually a spouse or adult child). All primary outcomes were measured at the 12^th^ month of the follow-up period after joining the study. Intra-individual comparison of therapeutic responses was conducted using two indicators: cognitive function (assessed using the MMSE) and global status (assessed using the CDR-SB). The patients with a second MMSE score equal to or higher than their first MMSE score (MMSE score ≥ 0) were considered as having a favorable response, whereas those with a lower second score were considered as having a poor response. Similarly, the patients with a second CDR-SB score lower than or equal to their first CDR-SB score (CDR-SB score ≤ 0) were considered as having a favorable response, whereas those with a higher score were considered as having a poor response. This study was approved by the Institutional Review Board of Kaohsiung Medical University Hospital (KMUHIRB-20140063), and written informed consent was obtained from all the patients or their legal representatives.

### ApoE genotyping

In all patients, the restriction enzyme iso-typing of the ApoE allele was performed following a modification of the protocol developed by PyrosequencerTM (http://www.pyrosequencing.com). Briefly, 10 ng of DNA was amplified in a 20-L reaction volume in which dGTP was replaced by a mixture of 25% dGTP and 75% dITP to facilitate analysis of the GC-rich fragment. A 276-bp fragment was generated using the forward primer AGA CGC GGG CAC GGC TGT and the reverse biotin-labeled primer CTC GCGGAT GGC GCT GAG. The single-stranded DNA prepared using streptavidin-coated beads and ApoE variants at codons 112 and 158 were pyrosequenced using the primers SNP112 GAC ATG GAG GAC GTG and SNP158CCG ATG ACC TGC AGA, applying the dispensation order GCTGAGCTAGCGT. Patients with one or two copies of the ApoE ε4 allele were considered ApoE ε4-positive, and those with no copy of the ApoE ε4 allele were considered ApoE ε4-negative.

### Statistical analysis

Data analysis was performed using the Statistical Package for Social Sciences software (Standard Version 11.5.0; SPSS Inc., Chicago, IL, USA). All statistical tests were 2-tailed, and an alpha value of .05 indicated significance. The *t* test was used to assess differences between the two independent groups (i.e., the favorable and poor response groups) regarding age, educational level, initial CDR-SB score, initial MMSE score, second CDR-SB score, and second MMSE score. The chi-squared test was used to compare the case–control and therapeutic groups with regard to the ApoE ε4 genotype and sex. In addition, the chi-squared test was used to compare the therapeutic groups with regard to cilostazol use.

Multiple logistic regression models were fit to the data to calculate the odds ratios (ORs) and 95% confidence intervals (CIs) of the association between the therapeutic response and cilostazol use. This model was adjusted for age, sex, educational level, initial MMSE score, initial CDR-SB score, and ApoE ε4 status.

The dependent variable in each logistic regression model was the response (favorable or poor), and either of the therapeutic indicators was examined separately. Independent variables, including age, educational level, initial CDR-SB score, and initial MMSE score, were treated as continuous variables with 1-year increments for age and educational level and 1-score increments for CDR-SB and MMSE scores. This contrasted with the dichotomous categorical variables including cilostazol use, sex, and ApoE ε4 status. The R squared for the logistic regressions is 31.12%. The lack of fit chi-squared is not significant (Prob > ChiSq = 0.2791).

## Results

The age, sex, educational level, first and second MMSE and CDR-SB scores, and ApoE genotyping results of all the particpants are presented in Table 1. Their average age, educational level, initial MMSE score, initial CDR-SB score, 12th-month MMSE score, and 12th-month CDR-SB score were 82.4 years, 7.8 years, 14.5, 8.1, 12.0, and 9.4, respectively, with no significant differences between the study and control groups. Of the patients, a majority were women (76.7%) and ApoE ε4-negative (78.2%), with no significant differences between the study and control groups.

**Table 1:** Baseline characteristics of case and control groups

	Case (N = 30)	Control (N = 30)	*p*-value
Age, years (mean ± SD)	82.8 ± 5.2	82.0 ± 5.9	0.580
Education, years (mean ± SD)	8.1 ± 4.3	7.6 ± 5.2	0.722
Gender, female (%)	21 (70.0)	25 (83.3)	0.222
1st MMSE	14.0 ± 6.5	14.9 ± 6.1	0.585
1st CDR_SB	8.8 ± 3.5	7.3 ± 3.1	0.103
2nd MMSE	11.5 ± 8.1	12.5 ± 7.3	0.617
2nd CDR_SB	9.9 ± 4.0	8.9 ± 3.4	0.290
ApoE ε4(+),n (%)	5 (16.7)	7 (23.3)	0.469

Case: participants using cilostazol for one year; Control: participants matching case participants 1:1 for age and education but not using cilostazol for one year

*MMSE* Mini-Mental Status Examination, *CDR_SB* Clinical Dementia Rating Sum of Boxes scale, *ApoE* apolipoprotein E

Table 2 shows factors associated with the therapeutic indicator of cognition. For the MMSE score, 31.7% of all participants had a favorable response, whereas 68.3% had a poor response. Moreover, significant differences were observed between the 2 therapeutic groups in cilostazol use (*P* = .015) but not in initial MMSE score, initial CDR-SB score, age, sex, educational level, or ApoE ε4 status (Table 2). However, after adjustment for other covariates in the logistic regression analysis, cilostazol use (OR = 0.17, 95% CI = 0.03–0.80, *P* = .024), initial CDR-SB score (OR = 2.06, 95% CI = 1.31–3.72, *P* < .001), and initial MMSE score (OR = 1.41, 95% CI = 1.11–1.90, *P* = .004), but not age, sex, education, or ApoE ε4 status, were significantly associated with poor therapeutic outcomes (Table 3).

**Table 2:** Therapeutic indicator of cognition: ΔMMSE

Therapeutic response, N (%)	Favorable^a^,21 (31.7)	Poor^b^,39(68.3)	*p*-value
Age, years (mean ± SD)	82.3 ± 5.6	82.5 ± 5.6	0.908
Education, years (mean ± SD)	7.5 ± 4.7	8.1 ± 4.8	0.634
Gender, female (%)	15 (71.4)	31 (79.5)	0.482
1st CDR-SB	7.4 ± 3.6	8.4 ± 3.3	0.292
1st MMSE	14.1 ± 7.1	14.6 ± 5.9	0.766
Cilostazol use			0.015
No	6 (20.0)	24 (80.0)	
Yes	15 (50.0)	15 (50.0)	
ApoE ε4(+),n (%)	7 (35.0)	5 (14.3)	0.074

*MMSE* Mini-Mental Status Examination, *CDR_SB* Clinical Dementia Rating Sum of Boxes scale, *ApoE* apolipoprotein E, ΔMMSE 2nd MMSE – 1st MMSE

^a^ΔMMSE ≥0

^b^ΔMMSE < 0

**Table 3:** Logistic regression for the therapeutic indicator of cognition: ΔMMSE

	odds ratio	95% CI	*p*-value
Age, years (mean ± SD)	0.96	0.82–1.13	0.645
Education, years (mean ± SD)	0.95	0.79–1.15	0.584
Gender, female (%)	2.23	0.34–16.02	0.401
1st CDR–SB	2.06	1.31–3.72	<0.001
1st MMSE	1.41	1.11–1.90	0.004
Cilostazol use	0.17	0.03–0.80	0.024
ApoE ε4(+), n (%)	0.30	0.04–1.90	0.3173

Odds ratios are based on comparing odds of poor (ΔMMSE < 0) versus favorable (ΔMMSE ≥0) therapeutic indicators of cognition

*MMSE* Mini-Mental Status Examination, *CDR_SB* Clinical Dementia Rating Sum of Boxes scale; *ApoE* apolipoprotein E, ΔMMSE: 2nd MMSE–1st MMSE

Table 4 shows factors associated with the therapeutic indicator of global status. For CDR_SB, 37.9% of all participants exhibited a favorable response, whereas 62.1% exhibited a poor response. No significant differences were observed between the 2 therapeutic groups in age, sex, educational level, initial MMSE score, or ApoE ε4 status (Table 4). In the logistic regression analysis, no significant association was observed between covariates and poor therapeutic outcomes (Table 5).

**Table 4:** Therapeutic indicator of global status: ΔCDR-SB

Therapeutic response, N (%)	Favorable^a^,22 (37.9)	Poor^b^,36 (62.1)	*p*-value
Age, years (mean ± SD)	82.1 ± 6.2	82.7 ± 5.3	0.741
Education, years (mean ± SD)	7.7 ± 5.1	7.9 ± 4.6	0.888
Gender, female (%)	14 (63.6)	30 (83.3)	0.089
1st CDR-SB	8.8 ± 3.6	7.6 ± 3.2	0.207
1st MMSE	13.8 ± 6.7	14.9 ± 6.1	0.534
Cilostazol use			0.104
Yes	14 (48.3)	15 (51.7)	
No	8 (27.6)	21 (72.4)	
ApoE ε4(+),n (%)	6 (31.6)	6 (17.7)	0.245

*MMSE* Mini-Mental Status Examination, *CDR_SB* Clinical Dementia Rating Sum of Boxes scale; *ApoE* apolipoprotein E, ΔCDR_SB: 2nd CDR_SB – 1st CDR_SB

^a^ΔCDR_SB ≤ 0

^b^CDR_SB > 0

**Table 5:** Logistic regression for the therapeutic indicator of global status: ΔCDR-SB

	odds ratio	95% CI	*p*-value
Age, years (mean ± SD)	1.01	0.88–1.15	0.920
Education, years (mean ± SD)	1.02	0.88–1.21	0.778
Gender, female (%)	3.33	0.68–18.62	0.146
1st CDR-SB	0.90	0.63–1.28	0.573
1st MMSE	1.00	0.82–1.22	0.962
Cilostazol use	0.51	0.12–1.99	0.331
ApoE ε4(+),n (%)	0.39	0.07–1.92	0.248

Odds ratios are based on comparing odds of poor (CDR_SB > 0) versus favorable (ΔCDR_SB ≤ 0) therapeutic indicators of global status

*MMSE* Mini-Mental Status Examination, *CDR_SB* Clinical Dementia Rating Sum of Boxes scale, *ApoE* apolipoprotein E, ΔCDR_SB: 2nd CDR_SB–1st CDR_SB

## Discussion

The main finding of this study is that cilostazol add-on therapy was associated with reduced odds of clinical deterioration of cognitive function for approximately 1 year in patients with stable AD who had received AChEIs for at least 12 months. In addition, we observed that high initial MMSE and CDR-SB scores were significantly associated with poor therapeutic responses in cognition. By contrast, cilostazol use, initial MMSE score, and initial CDR-SB score were not significantly associated with poor therapeutic responses in global status. Moreover, ApoE ε4 status and sex were not associated with therapeutic outcomes in cognition and functional status.

Cilostazol, an antiplatelet drug, improved cerebral circulation, reduced amyloid beta (Aβ) accumulation, and improved brain functioning in an experimental AD model [19]. A retrospective study reported that cilostazol improved cognitive function in patients with mild cognitive impairment [20]. Moreover, a pilot study of 10 patients with moderate AD conducted in a clinical setting reported that cilostazol reduced the rate of cognitive decline when co-administered with donepezil after an average follow-up period of 9.4 months [13]. In addition, cilostazol effectively suppressed cognitive decline in patients with AD and comorbid cerebro-vascular diseases [21] and in patients with mild dementia receiving donepezil, but did not have this effect in those with moderate and severe dementia [22]. In the current study, cerebral circulatory impairment and Aβ accumulation may have coexisted and varied significantly between patients. However, this situation may be representative of that generally observed in the elderly population, indicating the clinical relevance of administering a drug that has dual roles in ischemia and Aβ-induced neuro-degeneration. Thus, the preservation of cognitive function in patients with AD receiving a combination therapy of cilostazol and AChEIs may be clinically significant. One of the plausible mechanistic explanations for the positive effect of this combinatorial therapy is that AChEIs and cilostazol have different vascular targets. AChEIs increase acetylcholine levels, which in turn dilates vessels in an endothelium-dependent manner, whereas cilostazol targets PDE3 in the vascular smooth muscle cells and thus causes vasodilation in an endothelium-independent manner. Several “single-target, single-action” treatments for AD, such as antiamyloid agents, antioxidants, and anti-inflammatory drugs, have mostly failed or performed poorly in large clinical trials [23], leading to the complementary “neurovascular hypothesis.” [24] In AD, multiple pathogenic cascades originating from the altered vasculature can initiate the disintegration of the neurovascular unit, which can amplify Aβ deposition, as well as synaptic, neuronal, and glial dysfunction, and subsequent cognitive decline [25]. The current study suggests that vasoactive cilostazol may be a promising new therapeutic approach to maximizing the potential to improve cognitive function in patients with AD receiving AChEIs.

During the evaluation of therapeutic responses in global status, we observed that cilostazol use, initial global status, and initial cognitive function were not significantly associated with the worsening of global status, despite our patients already receiving AChEIs. These results differ from those of previous studies, which have reported that clinical deterioration is more common in the more advanced stages of dementia than in the early stages of dementia [26, 27]; moreover, patients in these studies were already receiving donepezil. Such findings may be partly due to different patients’ age and therapy strategies.

Our study has several strengths. First, unlike other studies, we evaluated the effect of the combination therapy of cilostazol and any AChEI, rather than only donepezil. Second, we controlled other variables, including age, sex, educational level, and ApoE ε4 status to have fewer confounding factors. Third, a previously published clinical trial obtained significant results by comparing the therapeutic effects of combination therapy (donepezil and cilostazol) with those of only donepezil therapy and by comparing mean differences in neurological measurements among groups. However, these results cannot be easily duplicated and applied in every patient with AD because not all patients respond to such treatments. We performed intra-individual comparisons to evaluate the therapeutic responses of patients with AD because, in real clinical settings, therapeutic responses are evaluated by comparing the current conditions of patients with their previous conditions.

Our study had some limitations that should be addressed. First, we used MMSE and CDR-SB, but not other neurological measurements, as therapeutic indicators, and these measurements may be insufficient for measuring the overall therapeutic response of patients with AD. Notably, previous studies have used changes from baseline MMSE [28] and CDR-SB [29] scores as therapeutic indicators. No consensus has been reached on the most efficient therapeutic parameters for reflecting the clinical condition of patients with AD receiving AChEIs. Second, we did not have detailed information on the type of AChEI used by each patient. Thus, we could not distinguish the effects of different AChEIs. However, the overall benefit of AChEIs remains limited [8].

Third, we did not consider the effects of concomitant medications, comorbid medical illnesses, or stroke. However, we conducted physical and neurological examinations in all the patients to detect any possible confounding factors. Moreover, according to Taiwan National Health Insurance rules and our study design, a minimum requirement is that patients with AD receiving AChEIs should undergo brain computed tomography to ensure no evidence of stroke as well as laboratory testing to rule out the presence other medical illnesses contributing to the AD diagnosis. Additional studies can be conducted to clarify the effect of these confounding factors. Finally, the proportion of apolipoprotein E (APOE) ε4 carriers in this study is relatively lower than that in other populations despite the similar proportion in previous studies in Taiwan. This may limit the possibility of external generalization.

## Conclusion

Our results indicate that cilostazol, which affects both cerebral circulation and Aβ metabolism, may reduce the odds of clinical deterioration of cognitive function in patients with stable AD. Because no fundamental treatment is available for AD, new therapies should be developed. Our results highlight the need for a comprehensive prospective cohort study to clarify the therapeutic response of cilostazol on the preservation of cognitive function in patients with AD.

## Abbreviations

AChEIs: Acetylcholinesterase inhibitors
AD: Alzheimer’s disease
Aβ: Amyloid beta
CASI: Cognitive assessment screening instrument
CDR-SB: Clinical dementia rating sum of boxes
MMSE: Mini-mental state examination
PDE3: Phosphodiesterase 3
